# IGF2BP1 is the first positive marker for anaplastic thyroid carcinoma diagnosis

**DOI:** 10.1038/s41379-020-0630-0

**Published:** 2020-07-27

**Authors:** Jacob Haase, Danny Misiak, Marcus Bauer, Nikolaos Pazaitis, Juliane Braun, Rebecca Pötschke, Alexander Mensch, Jessica Lilian Bell, Henning Dralle, Udo Siebolts, Claudia Wickenhauser, Kerstin Lorenz, Stefan Hüttelmaier

**Affiliations:** 1 Institute of Molecular Medicine, Section of Molecular Cell Biology, Martin Luther University Halle-Wittenberg, Charles Tanford Protein Center, Halle, Germany; 2 Institute of Pathology, Martin Luther University Halle-Wittenberg, Halle, Germany; 3 Department of General, Visceral and Transplantation Surgery, Section of Endocrine Surgery, University of Duisburg-Essen, Essen, Germany; 4 Department of Visceral, Vascular, and Endocrine Surgery, Martin Luther University Halle-Wittenberg, Halle, Germany; 5 Present Address: Merck KGaA, Darmstadt, Germany; 6 Present Address: Department of Neurology, Martin Luther University of Halle-Wittenberg, Halle, Germany

**Keywords:** Diagnostic markers, Thyroid cancer, Transcriptomics

## Abstract

Anaplastic thyroid carcinomas (ATC) are rare, but represent the most lethal malignancy of the thyroid. Selective molecular markers and drivers distinguishing ATC from other thyroid carcinomas of follicular origin remain largely unknown, limiting advances in diagnosis and treatment. In a retrospective study, we analyzed gene expression in 36 ATC, 18 poorly differentiated, 132 papillary, and 55 follicular thyroid carcinoma, as well as 124 paired and unpaired normal thyroid tissues in three independent cohorts by RNA-sequencing and immunohistochemistry. RNA-sequencing data in the test cohort suggested selective ATC protein biomarkers. Evaluation of these revealed that ATCs are characterized by the de novo expression of various testis antigens, including melanoma-associated antigen A3 (MAGEA3), but most importantly the oncofetal IGF2 mRNA binding protein 1 (IGF2BP1). Shallow whole genome sequencing essentially excluded that IGF2BP1 upregulation results from gene copy number alterations. Immunohistochemical analyses in all three tumor cohorts confirmed the selective de novo expression of IGF2BP1 protein in ATC. In sum, 75% (27/36) of all tested ATC and 0.5% (1/204) of poorly and well-differentiated thyroid carcinoma tissue samples were positive for IGF2BP1 protein. This indicates that IGF2BP1 protein expression identifies ATC with a diagnostic odds ratio of 612 (95% CI: 74.6–5021). In addition, we found that MAGEA3 is exclusively, although less consistently upregulated in ATC, presenting with an odds ratio of 411 (95% CI: 23.8–7098.7). Importantly, we provide confirmatory evidence that IGF2BP1 and MAGEA3 expression distinguishes ATC from poorly differentiated thyroid carcinoma. IGF2BP1 furthermore identified ATC foci within low-grade follicular thyroid carcinoma. In conclusion, IGF2BP1 represents the most promising single-gene marker available for ATC, followed by MAGEA3, improving on current techniques. Robust markers are essential to help distinguish this high-grade malignancy from other thyroid carcinomas, to guide surgical decision making, therapy and post-resection/therapy monitoring strategies.

## Introduction

Thyroid cancer of follicular origin is the most common endocrine malignancy, with significantly increasing incidence. The majority of tumors are further classified in well-differentiated carcinomas (WDTCs) including papillary thyroid carcinomas (PTC; incidence, 80–90%) and follicular thyroid carcinomas (FTC; incidence, 10–15%). These are distinguished from poorly differentiated (PDTC; incidence, 1–6%) and considerably rarer anaplastic thyroid carcinomas (ATC; incidence, 1–2%) [1, 2]. The majority of WDTCs have a good prognosis, and PDTC an intermediate prognosis. ATC, however, present a nearly uniformly fatal disease, accounting for the majority of thyroid cancer-associated deaths [3, 4].

Diagnosis of an ATC, either arising de novo or as aggravation of a WDTC or PDTC, demands for urgent and radical surgical interventions due to the local aggressive behavior and early metastasis formation, but still therapy options remain palliative. Therefore, the possible accurate identification, even of already microscopic foci of ATC within lower-grade thyroid cancer, would bring a benefit to any patient in sufficient time [1, 3, 5].

Although, histological classification of thyroid cancer remains the gold standard for diagnosis, massive-parallel sequencing results are increasingly being considered. Mutations in *BRAF*, *(H/K/N) RAS,* and other genes are found to a certain degree in all thyroid cancers of follicle epithelium, including ATC. However, these insights have failed to greatly improve patient survival [3, 6, 7]. An FDA-approved therapy option targeting BRAF-mutated ATC by applying dabrafenib and trametinib [8] changed the therapeutic landscape of the disease, but continued dismal outcomes highlight the need to identify selective markers and reveal targetable genes expressed in ATC. To further point at specific marker-driven approaches for ATC therapy and diagnosis improvement, the investigation of the ATC transcriptome should be instrumental. To date, microarrays and RNA-sequencing (RNA-seq) datasets on thyroid carcinomas are available [6, 9], but did not elucidate factors exclusive to ATC. Notably, ATCs are not included in TCGA transcriptomic datasets limiting avenues for biomarker and drug development.

The oncofetal IGF2 mRNA binding protein 1 (IGF2BP1) is a *bona fide* oncofetal protein, upregulated in some advanced solid cancers. The protein promotes the expression of oncogenes like MYC, LIN28B, and SRF by impairing their mRNA decay [10–12]. Consistent with its role supporting oncogene expression, IGF2BP1 was reported as a posttranscriptional driver of tumor cell proliferation, migration, metastatic potential, and therapy resistance [13, 14]. In that respect, it represents a prognostic marker for low survival probability in ovarian and neuroblastic cancers [15, 16].

Here, we reveal that IGF2BP1 and MAGEA3 are the first reliable protein and RNA markers of ATC, specifically distinguishing this malignancy from any other thyroid cancer of follicular origin, including PDTC. Besides their diagnostic value, therapeutic targeting of both may provide a promising future perspective for the treatment of ATC, independent of mutational status.

## Methods

### Patient samples

For the test cohort ten human primary ATC, six PTC and six FTC samples were collected from 1999 to 2012 at the University clinic of Halle, Germany. In addition, six nonmalignant thyroid tissue samples (NT) samples served as healthy controls. Specimens were formalin-fixed and paraffinized for immunohistochemistry, or snap frozen in liquid nitrogen and stored at −80 °C. All samples were re-evaluated histologically and with review of patient records by two pathologists (NP and CW).

An independent in-house tissue microarray (TMA I) contained 147 primary thyroid cancer samples (20 ATC, 18 PDTC, 82 PTC, and 29 FTC) and 108 paired normal thyroid tissue samples of all entities. A commercial microarray (TMA II, TH8010a, Biomax) contained 6 primary ATC, 44 PTC, 20 FTC, and 10 unpaired normal tissue samples.

The clinical characteristics of the tumor cohorts are summarized in Table 1.

**Table 1:** Clinical characteristics of tumor cohorts.

	No. of tumors (%)									
	RNA-seq (test cohort)			In-house tissue microarray (TMA I)				Commercial tissue microarray (TMA II)		
	ATC	PTC	FTC	ATC	PDTC	PTC	FTC	ATC	PTC	FTC
Total	10	6	6	20	18	82	29	6	43	20
Sex										
Female	7 (70)	3 (50)	4 (66.7)	10 (50)	10 (55.6)	44 (53.7)	11 (37.9)	4 (66.7)	37 (86.1)	14 (70)
Male	3 (30)	3 (50)	2 (33.3)	10 (50)	8 (44.4)	38 (46.3)	18 (62.1)	2 (33.3)	6 (13.9)	6 (30)
Median age, years (range)	68.5 (48–81)	50 (23–81)	55.5 (41–79)	67 (33–89)	70 (27–83)	47 (7–90)	63 (17–89)	55.5 (30–86)	40 (18–76)	54.5 (20–77)
UICC stage										
I	0	1 (16.7)	1 (16.7)	0	2 (11.1)	49 (59.8)	10 (34.5)	0	23 (53.5)	4 (20)
II	0	2 (33.3)	1 (16.7)	0	1 (5.6)	6 (7.3)	4 (13.8)	0	13 (30.2)	4 (20)
III	0	2 (33.3)	4 (66.7)	0	6 (33.3)	13 (15.9)	6 (20.7)	0	7 (16.3)	11 (55)
IV	10 (100)	1 (16.7)	0	13 (65)	9 (50)	13 (15.9)	1 (3.4)	6 (100)	0	1 (5)
nd	0	0	0	7 (35)	0	1 (1.2)	8 (27.6)	0	0	0

Data reported as No. (%), unless otherwise indicated.

*UICC* Union internationale contre le cancer.

### Immunohistochemistry

Immunohistochemistry was performed on 3 µm thick, consecutive sections of formalin-fixed, paraffin-embedded samples with the Bond Polymer refine detection Kit (Leica, DS9800), according to the manufacturer’s instructions on a fully automated immunohistochemistry stainer (Leica Bond). Sections were imaged with an Olympus BX50/51 microscope. Two pathologists (US and MB), independently and blinded to the clinical data, scored all samples by using a Histoscore, as described previously [17]. In brief, the relative amount of tumor cells being positively stained (%) was multiplied by their intensity from 0 (negative), 1 (weak), 2 (moderate), to 3 (intense). Expression classified into absent (0), low (1–100), intermediate (101–200), or strong (201–300) overall expression. Antibodies are summarized in Supplementary Table S2.

### Western blotting

For Western blotting, cells were lysed in lysis buffer (50 mM Tris-HCl (pH 7.4), 50 mM NaCl, 2 mM MgCl_2_, 1% SDS). Protein expression was analyzed by Western blotting with indicated antibodies (Supplementary Table 2), by an infrared scanner (LICOR).

### Databases

For Kaplan–Meier analysis, patient survival was analyzed by using the cBioPortal platform (http://cbioportal.org), combining patient data from the TCGA for PTC and FTC and from the MSKCC (Memorial Sloan Kettering Cancer Center) for ATC.

For the analysis of microarray data derived from Landa et al. (GEO Series accession number GSE76039; [6]), GEO2R was used.

### Deep-sequencing and differential gene expression

Total RNA was isolated from fresh frozen tissues by using the miRNeasy Kit (Qiagen), according to the manufacturer’s instructions. Total RNA-sequencing library preparation and sequencing was performed at the IKFZ (Leipzig, Germany). For total RNA-seq low-quality read ends as well as remaining parts of sequencing adapters were clipped using Cutadapt (v 1.4.2 or 1.6). Subsequently, reads were aligned to the human genome (UCSC GRCh19) using TopHat (v 2.0.12; [18]) or Bowtie2 (V 2.2.4; [19]), respectively. FeatureCounts (v 1.4.6; [20]) was used for summarizing gene-mapped reads. Ensembl (GRCh37.75; [21]) was used for annotations. Differential gene expression (DE) was determined by the R package edgeR (v 3.12.1; [22]) using TMM normalization, essentially as described previously [23].

Presented data have been deposited in NCBI’s Gene Expression Omnibus and are accessible through GEO Series accession number GSE126729. RNA-seq data are also available via the R2: Genomics Analysis and Visualization Platform (http://r2.amc.nl; datasets: “Tumor Thyroid Carcinoma – Huettelmaier”) for interactive use.

### GSEA analysis

Gene set enrichment analyses (GSEA) were performed as described previously [24]. The gene set hallmarks collection (H) was used for a list of all protein-coding genes ranked according to fold changes. The respective data were visualized by using the R package clusterProfiler [25].

### Statistics

Statistical analysis was performed using GraphPad Prism software (V7.0). Statistical significance was determined by using nonparametric Mann–Whitney test. Positive/negative predictive values (PPV, NPV) and diagnostic odds ratios (DOR) were determined by using MedCalc (V19.1.3). A principal component analysis was performed by using the R package pcaExplorer [26].

## Results

### IGF2BP1 is de novo expressed in ATC

**Fig. 1:**
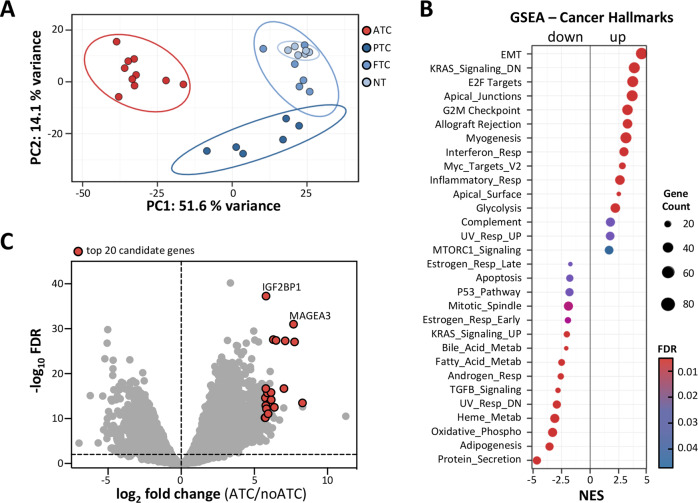
IGF2BP1 is de novo expressed in ATC. **a** Principle component (PC) analysis on RNA-seq data derived from the test cohort, including 10 ATC, 6 PTC, 6 FTC, and 6 NT samples. **b** Dot plot presentation of cancer hallmark gene sets upon a GSEA for differentially expressed genes for 10 ATCs vs 18 noATCs (6 PTC, 6 FTC, and 6 NT samples), investigated in (**a**). NES = normalized enrichment score. **c** Volcano plot of log_2_ mRNA fold changes plotted against the −log_10_ FDR (false discovery rate) for 10 ATCs versus 18 noATCs, investigated in (**a**). Horizontal dashed line indicates threshold (false discovery rate, FDR ≤ 0.01). Indicated in red are the top 20 ATC-exclusive genes, identified as described in the text.

The ATC is the most fatal thyroid malignancy (Supplementary Fig. S1a), but with the exception of few analyzed samples, comprehensive transcriptome analyses aiming to identify selective biomarkers are still rare [6, 9, 27, 28]. To identify novel markers of ATC, thyroid carcinoma gene expression was analyzed by RNA-seq in a test cohort (Table 1; clinical characteristics of the studied tumor cohorts). The protein-coding transcriptome of ten ATCs was compared with gene expression of six PTCs, six FTCs, and six NTs. A principle component analysis of transcriptome data illustrated that primary ATC samples cluster well together and are strikingly distinct from PTC, FTC and NT (Fig. 1a). In detail, a comparison of numbers on differentially expressed genes to NT revealed approx. 8000 differentially expressed genes (FDR ≤ 0.01) for ATC, but only approx. 500 and 100 for PTC and FTC, respectively (Supplementary Fig. S1b).

Cancer hallmark gene set enrichment analysis (GSEA) comparing ATC to PTC, FTC and NT, collectively referred to as “noATC,” dissected the molecular pathology aside frequently described mutations (Fig. 1b). These gene sets classify differentially expressed genes into well-defined hallmark pathways of cancer. Thus, essentially deregulated genes represented the invasive/pro-metastatic potential (epithelial-to-mesenchymal transition (EMT)), supported by dissociation of the apical junctional complexes, but also represented high rates of proliferation by ensuring fast cell cycle progression (G2M checkpoint; E2F targets). In support, genes directly activated by the MYC oncogene are upregulated, which also drive EMT and proliferation. ATCs are further distinguished by an increased expression of markers for inflammatory response and a severe alteration of metabolic processes, most prominently elevated glycolysis [29]. Pro-mesenchymal dedifferentiation of ATC was further supported by the reduced expression of thyroid markers like TSHR, epithelial markers like E-cadherin (CDH1) and the upregulation of stemness and EMT-associated markers MYC, SNAI2, TWIST1, OCT3/4 (POU5F1), LIN28B and NANOG (Supplementary Fig. S1c) [30–32]. Collectively, this indicated severe deregulation of the protein-coding transcriptome in ATC and suggested protein markers distinguishing this malignancy.

To identify exclusive protein markers of ATC, we assessed the de novo expression (mean FPM in noATC samples < 1; fold change in ATC against noATC samples > 50) of transcripts and evaluated the consistency of mRNA expression in ATC by ranking genes by increasing relative standard deviation (RSD) of expression in ATC (Fig. 1c; Table 2). The top 20-ranked de novo expressed protein-coding genes with low RSD of expression in ATC demonstrated the most consistent de novo expression for IGF2BP1 (Fig. 1c — top 20 genes in red). Interestingly, 12 of the 20 protein-coding genes, including IGF2BP1 and MAGEA (melanoma-associated antigen) proteins, are reported testis antigens [33]. These genes are of advanced interest in the focus of immunotherapy.

**Table 2:** Top 20 identified ATC-exclusive markers.

Gene name	log2 fold change (ATC vs noATC)	FDR (ATC vs noATC)	RSD	Cancer testis gene^a^
IGF2BP1	5.8008	4.8157E−38	0.1409	Yes
MAGEA2B	6.4879	4.3948E−28	0.3512	Yes
MAGEA2	6.3036	2.5740E−28	0.3526	Yes
DUX4L2	6.0914	3.3856E−21	0.3725	No
DUX4L6	5.7370	4.2742E−18	0.3737	No
DUX4L5	5.7618	4.7851E−17	0.3754	No
MAGEA3	7.6799	9.7494E−32	0.3813	Yes
MAGEA6	7.1070	5.0678E−28	0.4091	Yes
MAGEA12	7.7511	7.8765E−28	0.4806	Yes
DUX4	6.0547	1.4599E−18	0.4865	Yes
DUX4L3	5.7640	2.1113E−16	0.5032	No
XAGE1E	5.7851	2.0036E−17	0.6333	Yes
XAGE1B	5.9017	2.0458E−16	0.6573	Yes
XAGE1D	6.1376	1.7237E−16	0.7181	No
CT47A2	5.7574	2.6330E−15	0.7314	Yes
XAGE1A	7.0252	1.9515E−17	0.7458	No
CT47A6	5.9934	4.8163E−14	0.7730	Yes
MAGEC1	5.8336	6.3688E−13	0.8126	Yes
TMEM158	6.1610	6.0203E−15	0.8285	No
MSLN	7.0175	3.9930E−13	0.9407	No

*FDR* false discovery rate, *RSD* relative standard deviation.

^a^Information was obtained from the computational analysis by da Silva et al. [33].

As our test cohort did not include PDTC samples, mRNA expression was analyzed in an independent microarray dataset, comparing the transcriptomes of ATC and PDTC [6]. Reinvestigation of this study revealed that IGF2BP1 mRNA was reliably observed in 40% (8/20) of ATC samples. In sharp contrast, IGF2BP1 mRNA remained at background levels in all 17 PDTC samples included in the study (Supplementary Fig. S1d). Similar findings could be drawn for the MAGEA representative MAGEA3, with 40% (8/20) positive ATC and 5.9% (1/17) PDTC samples (Supplementary Fig. S1e). These findings provided independent support of the gene expression analysis in the here presented test cohort suggesting that IGF2BP1, but also MAGEA3, are selective markers of ATC to distinguish this malignancy even from PDTC.

**Fig. 2:**
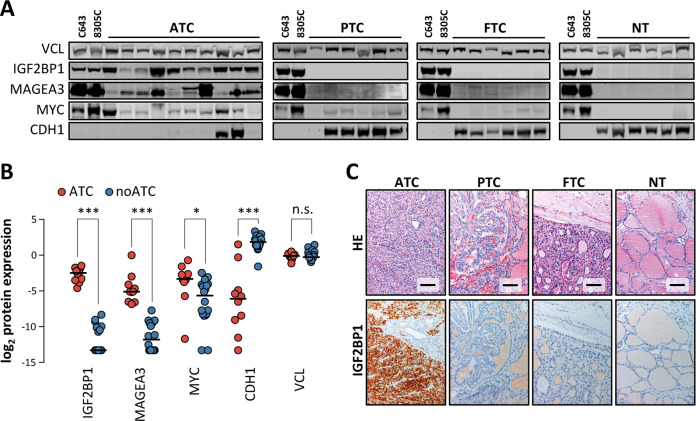
IGF2BP1 protein expression is detectable by Western blot and IHC in ATC samples. **a** Representative Western blot analysis of indicated proteins in two ATC-derived cell lines (C643 and 8305C) and protein lysates of individual samples analyzed by RNA-seq in (**a**). VCL served as loading control. **b** Scatter dot plot presentation of quantified log_2_ protein expression, determined for test cohort samples, investigated in (**a**). **c** IGF2BP1 expression analyzed by immunohistochemistry in representative samples investigated in (**a**). HE, hematoxylin eosin staining. Scale bars, 100 µm. Statistical significance was determined by Mann–Whitney test in (**d**) (***p ≤ 0.001; *p ≤ 0.05; n.s. not significant).

Exclusive expression of IGF2BP1 and MAGEA3 in ATC was initially validated by Western blotting in two ATC-derived cell lines (C643 and 8305C) and the individual samples of each thyroid cancer subtype comprised in the test cohort (Fig. 2a, b). The sharp and exclusive upregulation of both proteins in ATC samples was associated with enhanced expression of MYC, as well as the loss of CDH1 expression, confirming transcriptome studies. This suggested that IGF2BP1 provides a robust, positive marker for ATC at the mRNA as well as protein level. To evaluate this in further detail, representative tumor samples of the test cohort were analyzed by immunohistochemistry, confirming the selective de novo expression of IGF2BP1 protein in paraffinized ATC tissue (Fig. 2c; Supplementary Fig. S2). Importantly, in none of the other samples of the test cohort, IGF2BP1 protein expression was observed. This suggested IGF2BP1 as the first positive marker of ATC.

### IGF2BP1 de novo expression is unlikely a consequence of chromosomal aberrations

Recently, the *IGF2BP1* gene locus, located on the long arm of chromosome 17 (17q21.32), was found to be commonly gained and associated with poor survival probability in breast cancer and neuroblastoma [16, 34]. To further elucidate, whether alterations in copy numbers could be associated with de novo expression of IGF2BP1, shallow whole genome sequencing (sWGS) of the ATC samples from the initial test cohort was performed. However, copy numbers of *IGF2BP1* gene locus remained unchanged in 90% (9/10) of all ATC samples from the test cohort (Supplementary Fig. S3). Remarkably, in one tumor we detected a breakpoint at the *IGF2BP1* locus (sample #5).

### IGF2BP1 distinguishes ATC from other thyroid carcinoma of follicular origin

To investigate the potential use of IGF2BP1 as a diagnostic marker of ATC, two independent thyroid carcinoma validation cohorts, one previously assembled in-house tissue microarray (TMA I: 20 ATC, 147 tumor samples total and 108 paired NT samples) and a commercial tissue microarray (TMA II: 6 ATC, 70 tumor samples total, and 10 unpaired NT samples) were analyzed for IGF2BP1 protein expression by immunohistochemistry (Table 3). In addition, the analysis of MAGEA3, as a second promising marker, and MYC, as a well-known upregulated gene in high-grade thyroid carcinoma [35], was considered to be included into the study. IGF2BP1 protein expression, determined by Histoscores, was observed in 70% (14/20) of analyzed ATC in TMA I and 50% (3/6) in the TMA II (Fig. 2a, b; Supplementary Fig. S4a–c). Less stringent and consistent upregulation was also observed for MAGEA3 in 35% (7/20) ATC in TMA I, whereas expression in TMA II could only be detected in 16.7% (1/6) of ATC samples. Further, MYC expression was detectable in the majority of ATC samples (TMA I: 75%, 15/20; TMA I: 33.3%, 2/6). However, MYC expression was, to a lower extend, also observed in all other types of thyroid carcinoma, excluding this oncogene as a selective marker.

**Table 3:** Detectable expression of indicated proteins from the tumor cohorts.

	No. of tumors (%)									
	RNA-seq (test cohort)			In-house tissue microarray (TMA I)				Commercial tissue microarray (TMA II)		
	ATC	PTC	FTC	ATC	PDTC	PTC	FTC	ATC	PTC	FTC
Total	10	6	6	20	18	82	29	6	43	20
IGF2BP1	10 (100)	0	0	14 (70)	1 (5.6)	0	0	3 (50)	0	0
MAGE3A	10 (100)	0	0	7 (35)	0	0	0	1 (16.67)	0	0
MYC	9 (90)	5 (83.3)	3 (50)	15 (75)	2 (11.1)	1 (1.2)	5 (17.24)	2 (33.3)	0	0

Protein expression was detected by Western blot (samples prior used for RNA-seq) or by immunohistochemistry of tissue microarrays.

Importantly, IGF2BP1 protein expression could not be observed in any other tested thyroid tissue/tumor sample, except for 5.6% (1/18) of PDTC samples with a Histoscore < 100. MAGEA3 protein expression was detectable in 0% (0/18) and MYC in 11.1% (2/18) of all PDTC samples.

**Fig. 3:**
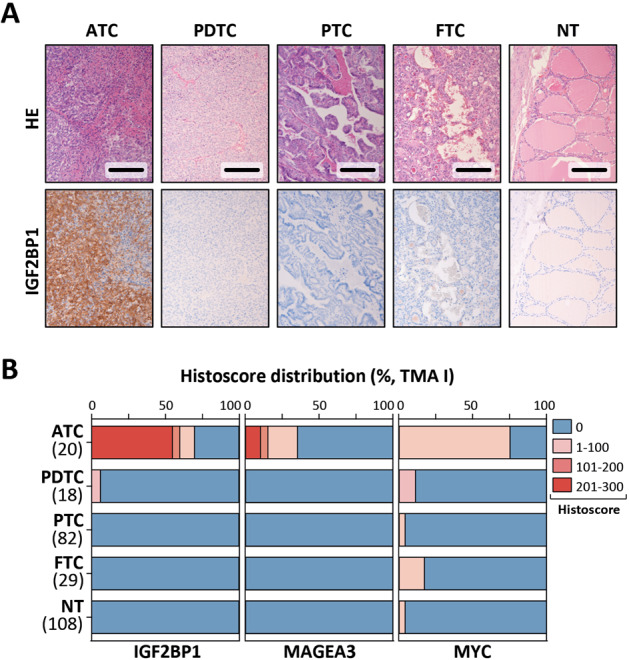
IGF2BP1 specifically identifies ATC of any other thyroid carcinoma of follicular origin by IHC. **a** IGF2BP1 expression analyzed by immunohistochemistry in representative samples derived from tissue microarray (TMA) I. HE, hematoxylin eosin staining. Scale bars, 100 µm. **b** Percentage view of IGF2BP1, MAGEA3 and MYC-histoscores for TMA I. Sample numbers are indicated.

To investigate IGF2BP1, but also MAGEA3 and MYC detection toward usability for diagnostic applications, we determined PPV and NPV, as well as DOR by testing with binary classification for the combination of the test cohort, TMA I and II. In sum, 75% (27/36) ATC samples were positive for IGF2BP1, whereas 5.6% (1/18) PDTC, 0% (0/132) of PTC and 0% (0/55) FTC samples revealed detectable IGF2BP1 protein expression. Diagnostic tests revealed overall PPV and NPV of ~100% and an exceptional overall DOR of 612 (95% CI: 74.6–5021) to identify ATC by IGF2BP1 detection (Fig. 3b, c). For MAGEA3 comparable PPV and NPV, but also a DOR of 411 (95% CI: 23.8–7098.7) were determined. Some detectable MYC protein expression in PDTC, PTC, and FTC led to a lower PPV of 61.9% (95% CI: 49.3–73.1) and a DOR of 30.7 (95% CI: 12.6–74.8). In conclusion, this indicated the potential use of IGF2BP1, but also MAGEA3, expression for discriminating ATC from other thyroid malignancies, including PDTC.

**Fig. 4:**
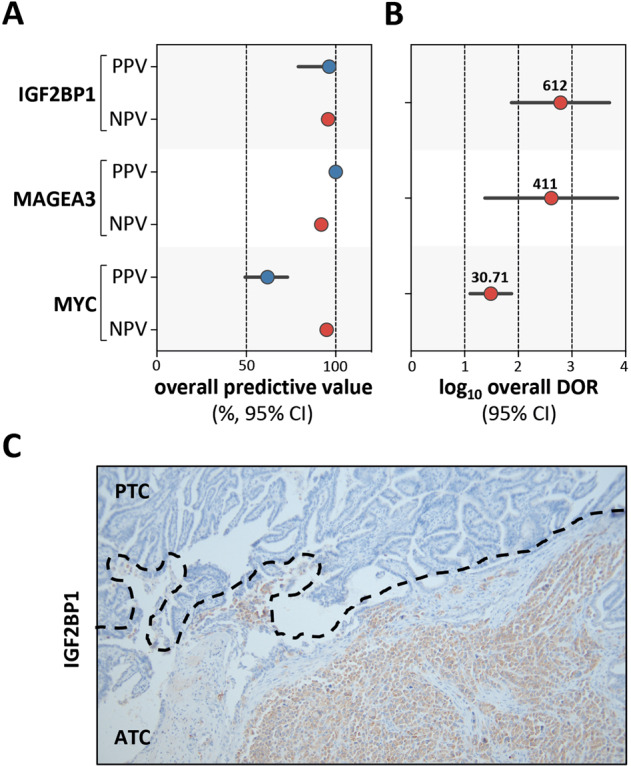
IGF2BP1 and MAGEA3 perform well as positive markers for ATC diagnosis. Plots of positive/negative predictive values (PPV/NPV) (**a**) and diagnostic odds ratio (DOR) (**b**) values for ATC diagnosis determined for indicated proteins by including all patient samples from the test cohort, tissue microarray I and II. Error bars indicate 95% confidence intervals (95% CI). **c** Detection of IGF2BP1 protein expression analyzed by immunohistochemistry on a patient-derived sample with ATC and PTC content.

This was strikingly supported by investigating IGF2BP1 protein expression in a patient-derived ATC sample with PTC content (Fig. 4c). In support of the ATC-selective expression of IGF2BP1, de novo expression was exclusively observed in the ATC area.

## Discussion

ATC is the most lethal malignancy of the thyroid, still lacking robust positive markers. In contrast to WDTC, the ATC is characterized by a rapid invasive growth, early metastasis and severe therapy resistance. Therefore, surgery in a limited stage is often the only potentially curative option [1, 2]. Aiming at a specific marker-driven approach to improve early ATC diagnosis, we combined a comparative RNA-seq analysis of distinct thyroid carcinomas of follicular origin and immunohistochemistry within a single methodological pipeline. This revealed robust and exclusive de novo expression of IGF2BP1, providing the first positive marker of this malignancy suitable for diagnosis on the mRNA and protein level.

Comparative transcriptome analyses and a subsequent cancer hallmark GSEA clearly dissected the molecular pathology of this distinct thyroid carcinoma from other subtypes, besides its frequently described mutations [7]. The GSEA outlines the molecular causes of histologic features for high rates of proliferation, but also an invasive behavior and in sum its high grade of dedifferentiation from its thyroid origin.

Further, the RNA-seq indicated de novo expression of testis antigens including MAGEA3 and IGF2BP1 as outstanding markers of ATC.

By sWGS we demonstrated that the de novo expression of IGF2BP1 in ATC can not be explained by gene gain, as previously observed in other tumors [16, 34]. Copy numbers of the *IGF2BP1* gene locus remained unchanged in 90% (9/10) amongst ATC samples. But the here presented observation fits previous reports on copy number alterations in ATC, showing that the chromosomal region 17q21 is not frequently gained or lost [7, 9, 36]. This suggested that the de novo expression of IGF2BP1 in ATC largely results from epigenetic and/or transcriptional deregualtion.

In two independent TMAs immunohistochemistry analyses demonstrated that the majority of ATC samples were IGF2BP1 positive. This rate likely will be improved by optimizing the sensitivity of immunostaining, since IGF2BP1 mRNA expression was observed in all analyzed ATC from the initial test cohort. IGF2BP1 also performed well against MAGEA3 and MYC, for which less pronounced intensities were observed in fewer ATC and also some WDTC samples. Supporting a differential ATC diagnosis from PDTC, only 5.6% (1/18) of PDTC samples identified with a low IGF2BP1 histoscore by immunohistochemistry. Its mRNA expression was completely absent in an independent microarray analysis. Comparable findings were made in terms of MAGEA3 detection.

Accordingly, the protein detection of IGF2BP1 and MAGEA3 appeared to be suitable for the diagnosis of ATC with exceptional results from diagnostic tests, including PPV, NPV, and DOR. Finally MYC immunoreactivity could be excluded to be useful for differential diagnosis of ATC, although it was recently reported to correlate with dedifferentiation in thyroid neoplasias [35]. Still, IGF2BP1 revealed the highest consistency.

Further, IGF2BP1-positive samples identified in TMA I showed distinct WDTC/PDTC or just ATC content (Supplementary Table S1). Thus, we can largely exclude that IGF2BP1 expression in ATC is dependent on the disease origin. Nevertheless, our study stresses that IGF2BP1 immunohistochemistry has the potential to help not only defining a diagnosis but also to identify early dedifferentiation in areas of solid histoarchitecture in case of WDTC or even PDTC to prevent underestimation of tumor severity.

Notably, the histological diagnosis of ATC can be challenging due to heterogeneous histological appearance and similarity to cancers like the undifferentiated lung adenocarcinoma, lymphoma, or thyroid angiosarcoma [1, 37]. One should consider that IGF2BP1 was reported to be de novo expressed in several high-grade malignancies [14, 38]. Thus, IGF2BP1 detection could potentially lead to false-positive diagnosis in the case of a rarely observed lung cancer-derived metastasis to the thyroid [1, 39]. Nonetheless, established markers for immunostaining of ATC samples are cytokeratins and the only retained thyroid-specific transcription factor PAX8. These markers can often present with weak and focal immunoreactivity [1, 40]. Thus, positive markers will, again, help defining a diagnosis.

In view of the probably mutation-independent and sharp upregulation of IGF2BP1 in ATC, as well as its frequently reported expression in other aggressive cancers [14, 38], our study strongly suggests expediting the clinical evaluation and also improvement of IGF2BP1-directed inhibitors in cancer therapy. In this respect, the IGF2BP1-specific inhibitor BTYNB has recently been developed, which could prove promising in preclinical investigations [41].

Interestingly, we identify MAGEA proteins, including MAGEA3, which is induced in a variety of metastatic cancers and has been targeted most recently in a phase-II clinical trial [42]. However, it failed in an extensive phase-III clinical trial in immunotherapy, but remains as a promising candidate for novel targeted treatment opportunities of ATC [43].

Conclusively, our study provides new insights into the whole transcriptomic landscape of this highly aggressive neoplasia, which is distinct from other thyroid carcinomas, besides its frequently reported characteristic mutational burden, including TP53 or TERT-promoter mutations [1, 6, 7]. In consequence IGF2BP1, but also MAGEA3 are promising new candidates for fast clarification of disease severity, other than the established markers, although the here presented data requires additional confirmation by staining of whole sections and the conduction of extended studies in future.

## Supplementary information

Supplementary Figures

Supplementary Tables
