# Supplementary material for: IGF2BP1 is the first positive marker for anaplastic thyroid carcinoma diagnosis
Source: Mod Pathol. 2020 Jul 27;34(1):32–41. doi: 10.1038/s41379-020-0630-0 (PMC7806508; doi:10.1038/s41379-020-0630-0)
Supplement: Supplementary file 1 — Supplementary Figures [file 41379_2020_630_MOESM1_ESM.pdf]

SUPPLEMENTARY FIGURES

Figure S1

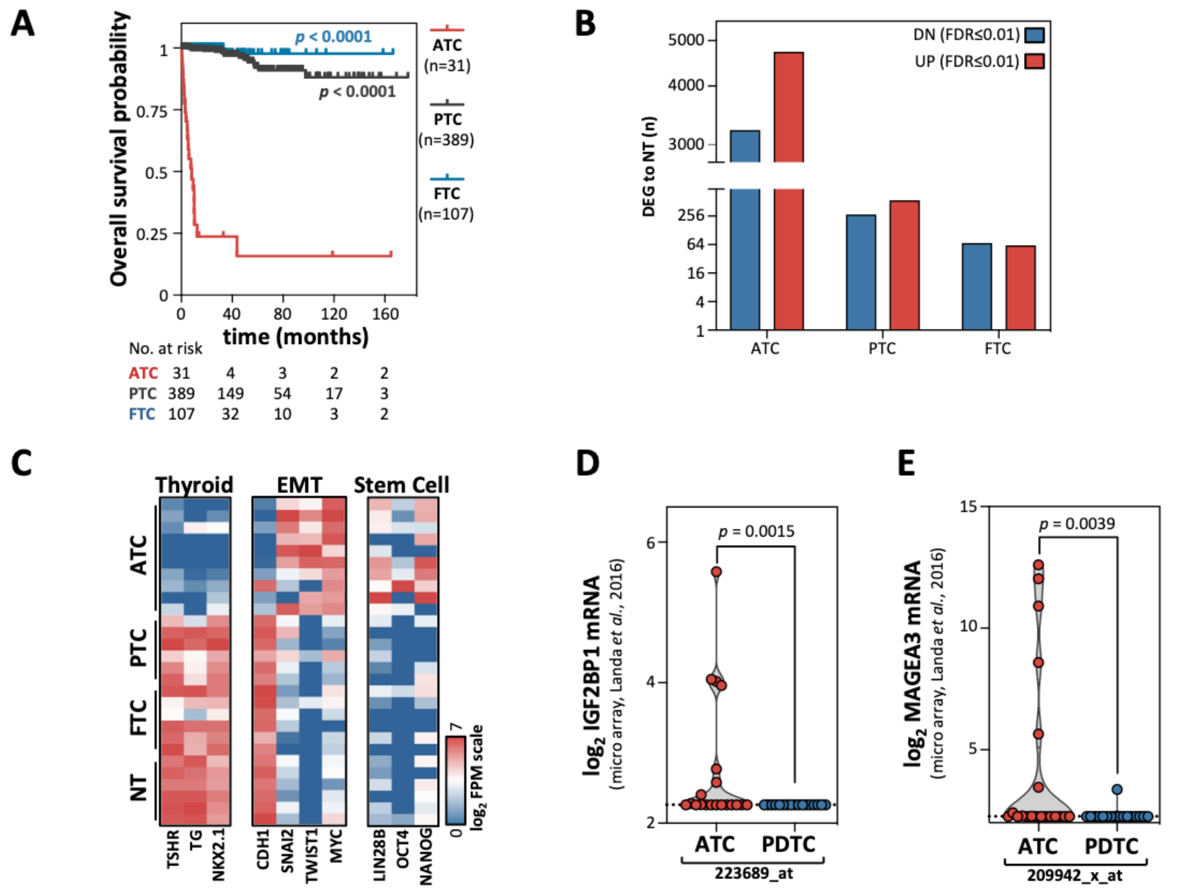

Figure S1. IGF2BP1 is *de novo* expressed in ATC. **A)** Kaplan-Meier analysis of overall survival data from PTC (n = 389), FTC (n = 107) and ATC (n = 31) patients. Data were derived from cBIO-portal (TCGA & MSKCC data). **B)** Number of differentially expressed (DEG; FDR ≤ 0.01) protein-coding genes (mRNA) for each thyroid carcinoma subtype versus NT, by RNA-seq. **C)** Heatmap presentation of log_2 FPM (fragments per million) values determined for indicated genes by RNA-sequencing (RNA-seq) in 10 ATC, 6 PTC, 6 FTC and 6 NT samples. **D, E)** Violin plot presentation of MAGEA3 and MYC mRNA (probe-identifier indicated) expression in 20 ATC and 17 PDTC samples. Horizontal dashed line indicates the micro-array-

dependent noise threshold. Statistical significance was determined by Log-rank-test (Mantel-Cox) in (A) and Mann-Whitney-test in (D, E).

Figure S2

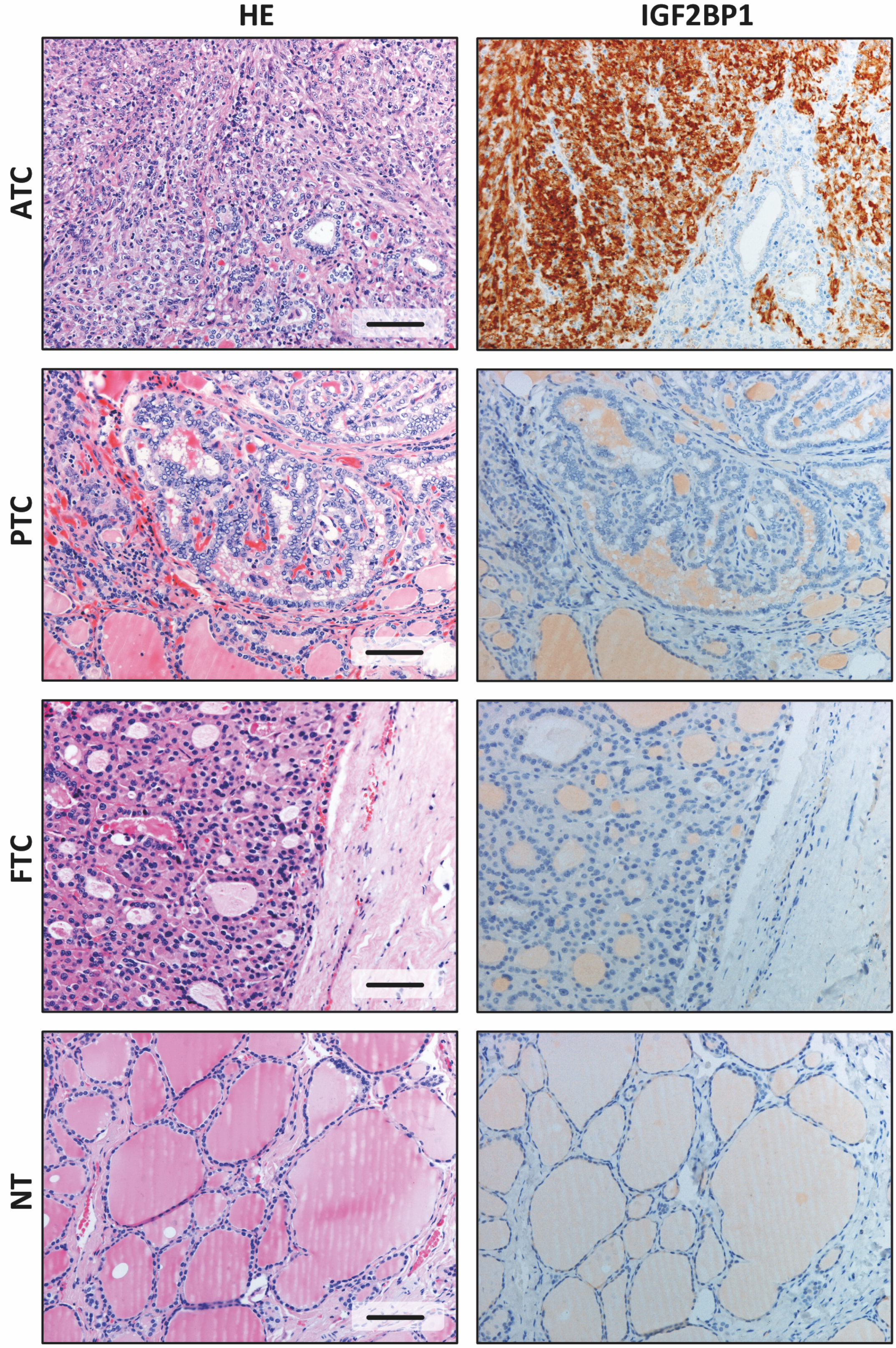

Figure S2. IGF2BP1 expression analyzed by immunohistochemistry on representative samples, as in Figure 2C. HE, hematoxylin eosin staining. Scale bars, 100 µm.

Figure S3

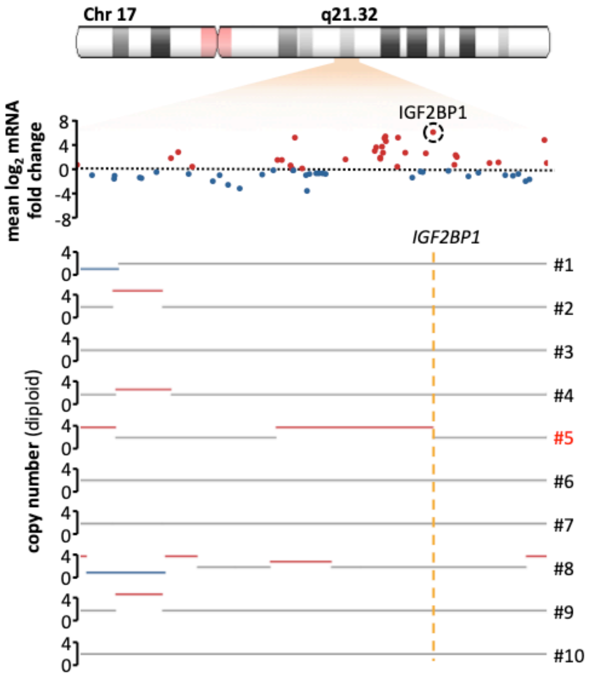

Figure S3. F) Genomic deletion or amplification events were determined by shallow Whole Genome Sequencing (sWGS) of ATC samples, prior used for RNA-seq (see Fig.1A). (Upper panel) Mean log2 mRNA fold changes and (lower panel) gene copy numbers of analyzed ATC samples are shown within the indicated region of chromosome 17. Blue lines indicate regions of copy number loss, red lines indicate regions of copy number gain. The position of the IGF2BP1 (17q21.32, dashed line) locus is highlighted.

Figure S4

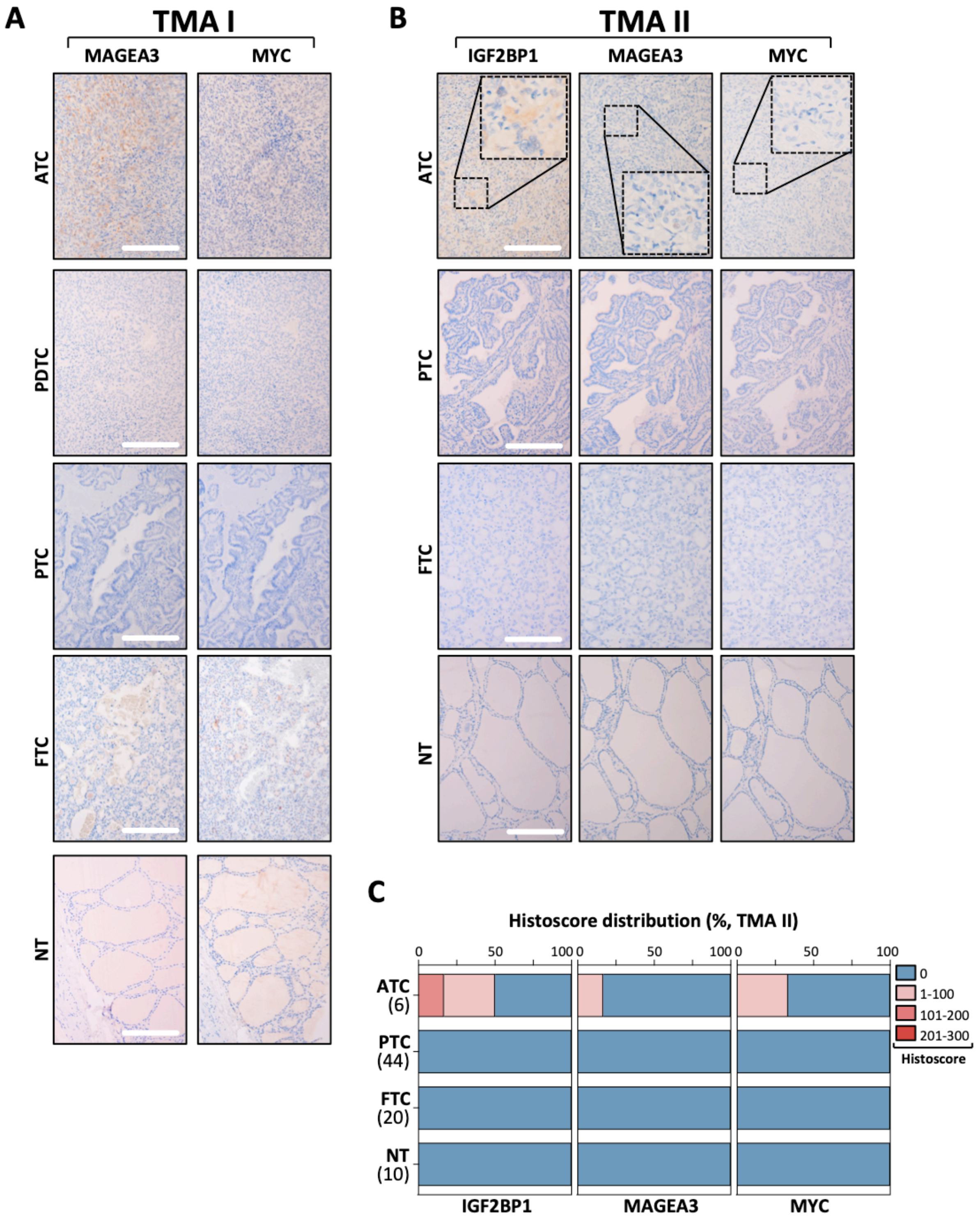

Figure S4. Representative tissue microarray cores. **A)** Representative tissue microarray cores investigated in Figure 2A for MAGEA3 and MYC expression analyzed by immunohistochemistry (TMA I). HE, hematoxylin eosin staining. Scale bars, 100 µm. **B)** Representative tissue microarray cores for IGF2BP1, MAGEA3 and

MYC expression analyzed by immunohistochemistry (TMA II). Insets = 2x magnification. Scale bars, 100 µm. **C)** Percentage view of IGF2BP1, MAGEA3 and MYC-Histoscores for TMA II. Sample numbers are indicated.
