# Supplementary material for: IGF2BP1 is the first positive marker for anaplastic thyroid carcinoma diagnosis
Source: Mod Pathol. 2020 Jul 27;34(1):32–41. doi: 10.1038/s41379-020-0630-0 (PMC7806508; doi:10.1038/s41379-020-0630-0)
Supplement: Supplementary file 2 — Supplementary Tables [file 41379_2020_630_MOESM2_ESM.pdf]

Supplemtary Table 1
WDTC-content TMA I

sample #	entity	IGF2BP1 protein	WDTC/PDTC content
1	ATC	pos	-
2	ATC	neg	-
3	ATC	neg	-
4	ATC	pos	PTC
5	ATC	pos	PTC
6	ATC	pos	-
7	ATC	pos	-
8	ATC	pos	PDTC
9	ATC	pos	-
10	ATC	neg	-
11	ATC	pos	-
12	ATC	pos	FTC + PDTC
13	ATC	pos	PDTC
14	ATC	neg	-
15	ATC	neg	-
16	ATC	pos	-
17	ATC	pos	-
18	ATC	pos	-
19	ATC	pos	-
20	ATC	neg	-

Supplementary Table 2
Materials - Antibodies

name	company	cat. no. #	RRID
anti-IGF2BP1 (WB)	BSBS AB facility	clone 6A9, previously described by Stöhr *et al.*, 2012	N/A
anti-IGF2BP1 (IHC)	MBL	RN001	AB_1953026
anti-MYC (WB)	Merck Millipore	06-340	AB_310106
anti-MYC (IHC)	Cell Marque	395R	N/A
anti-MAGEA3 (WB/IHC)	MERCK Millipore	MABC1150	N/A
anti-CDH1 (WB)	Abcam	ab40772	AB_731493
anti-VCL (WB)	Sigma-Aldrich	V9131	AB_477629
